# Impact of neuroendocrine neoplasm-specific systemic treatments on expression and function of CXCR4 in neuroendocrine tumor cells

**DOI:** 10.1038/s41598-026-37026-8

**Published:** 2026-01-31

**Authors:** Christof Däubler, Clara Böttcher, Laura-Sophie Landwehr, Alexander Meining, K. Michalski, F. Reiter, Rudolf A. Werner, Philipp Hartrampf, Dorothee Rogoll, Alexander Weich

**Affiliations:** 1 Department of Internal Medicine II, Division of Gastroenterology, University Hospital Würzburg, Oberdürrbacher Str. 6-8, 97080 Würzburg, Germany; 2 Department of Internal Medicine I, Division Endocrinology, University Hospital Würzburg, Würzburg, Germany; 3 Department of Nuclear Medicine, University Hospital Würzburg, Würzburg, Germany; 4 Department of Internal Medicine II, Division of Hepatology, University Hospital Würzburg, Würzburg, Germany; 5 Department of Nuclear Medicine, LMU University Hospital, LMU Munich, Munich, Germany

**Keywords:** Neuroendocrine neoplasm, CXCR4, Cisplatin, Everolimus, Temozolomide, Systemic therapy, Biomarkers, Cancer, Oncology

## Abstract

As neuroendocrine neoplasms (NEN) undergo increasing dedifferentiation, CXC chemokine receptor type 4 (CXCR4) becomes upregulated. Higher levels of CXCR4 are associated with invasive growth, metastasis formation, and poor prognosis. CXCR4 is considered a potential diagnostic biomarker and a promising target for precision therapies — e.g., endoradiotherapeutic approaches, monoclonal antibodies, or small-molecule inhibitors. A variety of systemic therapies are used to treat metastatic NEN, which may modulate CXCR4 expression and potentially influence the efficacy of future CXCR4-targeted strategies. In the NEN cell lines BON-1, QGP-1, and MS-18, we applied cisplatin, etoposide, streptozotocin, 5-fluorouracil, temozolomide, and everolimus- all systemic agents used in highly proliferative NEN. Following incubation, CXCR4 expression was quantified by qRT-PCR, Western blot, and immunohistochemistry. The functional receptor activity was determined by measuring uptake of the radioligand [68Ga]Pentixafor. Cisplatin induced a significant reduction in CXCR4 mRNA levels in BON-1 and QGP-1 cells (*p* < 0.05) and decreased radioligand uptake in QGP-1 and MS-18. Etoposide, 5-FU, and streptozotocin had no significant impact on CXCR4 expression or uptake activity. Temozolomide and Everolimus markedly diminished both CXCR4 mRNA and protein levels with no significant impact on radioligand uptake. In high-grade NEN cell lines, Cisplatin, Everolimus, and Temozolomide substantially diminish CXCR4 expression, with Cisplatin significantly decreasing CXCR4-targeted radioligand uptake. These findings might have an impact on the optimal therapy sequence and patient selection for future CXCR4-targeted approaches. Further, the decreased CXCR4 expression could represent a new mechanism of action of the established drugs Cisplatin, Temozolomide, and Everolimus.

**Supplementary Information:**

The online version contains supplementary material available at 10.1038/s41598-026-37026-8.

## Introduction

Neuroendocrine neoplasms (NEN) are rare neoplasms with a globally rising incidence. Although they originate from the neuroendocrine system, NENs are a heterogeneous group concerning primary site, differentiation, and proliferation. Approximately two-thirds of NENs develop in the gastrointestinal tract, especially in the ileum and the pancreas^1^.

NENs can be classified into well-differentiated neuroendocrine tumors (NETs) and dedifferentiated neuroendocrine carcinomas (NECs). The Ki67 proliferation rate ranges from below 1% in comparably benign NET G1 to over 20% in more aggressive NET G3^2^. Despite dedifferentiation, the neuroendocrine origin remains evident through functional properties and histological characteristics^3^. One example is the tissue-specific expression of somatostatin receptors, with somatostatin receptor type 2 (SSTR2) being the most clinically relevant subtype. SSTR2 plays a pivotal role in diagnosis via imaging modalities, such as DOTATOC PET/CT, and in the treatment of low-proliferative NETs. However, under therapy, further dedifferentiation can lead to the loss of SSTR2 expression^4–6^. NECs are typically dedifferentiated from neuroendocrine tissue and exhibit higher proliferation rates, with Ki67 indices often exceeding 80% and lacking SSTR2 expression^7,8^. Given that SSTR2 is the most important diagnostic and therapeutic target, alternative targets are urgently needed for dedifferentiated NENs.

In contrast, the expression of C-X-C motif chemokine receptor 4 (CXCR4) is associated with higher rates of dedifferentiation in NENs, increased locoregional aggressiveness, the emergence of metastases, elevated proliferation rates indicated by higher Ki67 indices, and consequently, a poorer prognosis^4,6,9^. CXCR4, along with its ligand CXCL12, plays an important role in hematopoiesis and embryogenesis. Its relevance in both hematological and solid malignancies has garnered attention in recent years^10^. A growing body of evidence supports CXCR4 as a promising target for a wide range of therapeutic and diagnostic applications. Several antagonists have been developed, including small molecules, modified peptides, antibodies, and microRNAs^11^. In a mouse model of non-small cell lung cancer (NSCLC), a direct CXCR4 inhibitor demonstrated a reduction in both the number and size of pulmonary metastases. Meanwhile, the development of monoclonal antibodies targeting CXCR4 in NENs is currently in the early stages^10,11^. In late-stage pancreatic cancer, CXCR4 inhibition combined with the checkpoint inhibitor pembrolizumab has shown promising results by enhancing CD8^+^ effector T cell infiltration into tumors^12^. Such synergistic effects may also be observed and leveraged in CXCR4-positive NENs.

The in vivo expression of CXCR4 can be visualized and monitored non-invasively using [^68^Ga]Pentixafor PET/CT. This technology facilitates a theranostic approach, enabling CXCR4-directed endoradiotherapy with agents such as [^177^Lu]/[^90^Y]Pentixather. An inverse relationship has been observed wherein increasing dedifferentiation in NENs correlates with loss of SSTR2 expression and a concomitant rise in CXCR4 expression^4,6,13^. The potential of CXCR4-directed radioligand therapy was shown in an animal xenograft model of pulmonary neuroendocrine cancer, where [^177^Lu]Pentixather effectively inhibited tumor growth and increased overall survival^14^.

In developing CXCR4-targeting diagnostic and therapeutic approaches for NENs, it is crucial to understand how other NEN-specific systemic therapies might influence CXCR4 expression. Notably, pharmacological modulation of the Wnt/ß-Catenin pathway has been shown to alter CXCR4 expression levels^15^.

There are six primary systemic therapies employed in the treatment of NENs lacking SSTR2 expression. In pancreatic NETs, temozolomide and capecitabine, a prodrug of 5-Fluorouracil (5-FU), are utilized, as well as streptozotocin in combination with 5-FU. The mTOR inhibitor everolimus is applicable for NET G3 of both the ileum and pancreas. For NECs, first-line therapy typically involves a combination of carboplatin, a platinum derivative, with the topoisomerase inhibitor etoposide^5^.

Given the absence of data regarding the influence of these substances on CXCR4 expression, we investigated their effects on two dedifferentiated NET cell lines, as well as on a primary NEN cell line exhibiting an intermediate phenotype characterized by abundant CXCR4 expression - a marker of dedifferentiation – while concurrently retaining tissue-specific SSTR2 expression^16^.

## Materials and methods

### Cell culture

The BON-1 and QGP-1 cell lines, both derived from human pancreatic NETs, were generously provided by Prof. C. Grötzinger from the Department of Hepatology and Gastroenterology, Charité-Universitätsmedizin in Berlin, Germany. Both cell lines were tested and confirmed to be free of mycoplasma contamination (Minerva biolabs, Berlin, Germany: Cat.No: 11–8025). To verify cell line identity, PCR-based single-locus technology using the PowerPlex 21 Kit (Promega) was employed, with subsequent analysis performed by Eurofins (Ebersberg, Germany). For QGP-1, identity was further confirmed by the Leibniz Institute (DSMZ) through short tandem repeat (STR) profiling. Although no reference STR profile for BON-1 exists in the DSMZ database, STR analysis revealed a consistent genetic profile without evidence of cross-contamination, in agreement with previously published data^8^. The MS-18 cell line, derived from a metastatic NEC of the rectum, was established from a patient biopsy. A molecular characterization of this cell line has been submitted for publication by our group^16^.

QGP-1 cells were cultured in RPMI-1640 medium (Gibco, Carlsbad, USA: Cat.No: 21875-034) supplemented with 10% fetal bovine serum (FBS Superior, Biochrom, Berlin, Germany: Cat.No: S06115) and penicillin/streptomycin (Pen/Strep; 1 × 10^5^ u/lM; Gibco, Carlsbad, USA, Cat.No: 15140-122). BON-1 cells were maintained in Dulbecco´s Modified Eagle´s Medium with nutrient mixture F12 Ham (Sigma Aldrich, Darmstadt, Germany: Cat.No: N4888), also supplemented with 10% FBS and Pen/Strep (1 × 10⁵ U/L). Both cell lines were cultured in 75 cm² culture flasks (Greiner Bio-One B.V., Kremsmünster, Austria) at 37 °C in a humidified incubator with 5% CO₂. Cells were passaged upon reaching confluence using 0.05% trypsin-EDTA (Gibco, Carlsbad, USA, Cat.No: 25300-054).

MS-18 cells were grown in T75 primary tissue culture flasks (Corning, NY, USA) using DMEM/F12 medium (Gibco, Carlsbad, USA, Cat.No: 11330-032), supplemented with 3% Nu-serum (Corning, NY, USA, Cat.No: 355100) and 1% insulin-transferrin-selenium (ITS) (Gibco, Carlsbad, Cat.No: 41400-045). These cells were also maintained at 37 °C in a 5% CO₂ humidified incubator and passaged with Accumax solution (Thermo Fisher; Cat.No. 00–4666-56) once confluent. Media for all three cell lines were refreshed every 3–4 days, with cells maintained for a maximum of 30 passages.

Cells were treated separately with the following agents: everolimus (Selleckchem, Houston, TX, USA; S1120), 5-floururacil (5-FU; Sigma Aldrich; F6627) for 48 h; cisplatin (Cipla, Mumbai, India), etoposide (Sigma Aldrich; E1383) and streptozotocin (STZ; ENZO Life Sciences, Inc., Plymouth Meeting, PA, USA; ALX-380-010-M100) for 24 h, and temozolomide (Selleckchem; S1237) for 96 h.

### Cell viability

The half-maximal effective concentration (EC_50_) values for growth inhibition by the tested compounds were determined using the CCK8 assay (Sigma Aldrich; Darmstadt, Germany: Cat.No. 96992). Cells were seeded in 96-well plates (Sarstedt, Nümbrecht, Germany) at a density of 7.500 cells per well. Each compound was applied at ascending concentrations and for varying incubation periods, in accordance with the manufacturer´s guidelines for in vitro research. The BON-1 and QGP-1 cell lines exhibit comparable sensitivity to the tested substances. In contrast, the MS-18 cell line demonstrated higher susceptibility, necessitating an adjustment of drug concentrations for this cell line. Consequently, while the same concentrations were used for BON-1 and QGP-1 due to their similar EC_50_ values, reduced concentrations were applied to MS-18 cells. Incubation times remained consistent across all three cell lines. The concentrations and incubation times used for BON-1 and QGP were as follows: cisplatin 100µM, etoposide 300µM, streptozotocin 2,5mM, 5-FU 750µM, and everolimus 100nM for 48 h, temozolomide 100µM for 96 h. For the MS-18 cell line, the following concentration and incubation times were applied: cisplatin 10µM for 48 h, etoposide 25µM for 24 h, streptozotocin 0.75 mM for 24 h, 5-FU 150µM for 48 h, temozolomide 10µM for 96 h, and everolimus 100nM for 48 h. The selection of concentrations was based on previously published data^16^.

### RNA isolation and quantitative RT-PCR

Quantitative reverse transcription PCR (qRT-PCR) was performed as previously described^17^. Briefly, total RNA was extracted using the NucleoSpin RNA kit (Machery-Nagel, Düren, Germany; Cat.No: 740955.250), and cDNA was synthesized from 1 µg of RNA using the iScript cDNA Synthesis Kit (BioRad, Hercules, CA, USA; Cat.No: 1708891) according to the manufacturer’s instructions. qRT-PCR was carried out using the Absolute QPCR Mix (Thermo Scientific, Waltham, MA, USA; Cat.No: AB1132/B), and gene expression was detected using the AB ViiATM7 Real-Time PCR System (Life Technologies, Carlsbad, CA, USA). TaqMan gene expression assays and endogenous controls were purchased from Applied Biosystems (Foster City, CA, USA, Cat.No for CXCR4: Hs00607978_s1; Cat.No for GAPDH: VIC/TAMRA #4310884E).

### Immunohistochemistry

CXCR4 expression in BON-1, QGP-1, and MS-18 cells was evaluated via immunohistochemistry. Cytospins were prepared by centrifuging 5 × 10⁵ cells per slide. A CXCR4-specific primary antibody (Abcam, Cambridge, UK, Cat.No: ab124824) was applied at a 1:200 dilution using the super-sensitive streptavidin–peroxidase antiperoxidase method (Biogenex, Fremont, CA, USA, Cat.No. QP900-9LE).

Slides were analyzed for CXCR4 expression under both control conditions and following drug treatment. The HeLa (ATCC, Manassas, VA, USA; CCL-2-1) cell line is a verified positive control for CXCR4 (as indicated by Abcam, Cambridge, UK). However, for methodological validation, it was used as both positive and a negative control by including and omitting the primary antibody, respectively, to confirm staining specificity and rule out contamination. Microphotographs were taken with EVOS M5000 equipped with EVOS Objective (40x).

### Western blot

Western blot analysis was conducted as described previously^18^. Cells were seeded at density of 5 × 10^5^ cells per well in 6-well plates (Greiner Bio-One B.V., Kremsmünster, Austria) and treated with the respective drugs, followed by incubation at 37 °C in a humidified atmosphere containig 5% CO_2_. After treatment, cells were washed twice with Dulbecco’s phosphate-buffered solution (DPBS, Gibco, Carlsbad, USA, Cat.No: 14190-169), and lysed in ice-cold RIPA buffer (Thermo Scientific; Cat.No: 89900) supplemented with a protease inhibitor cocktail (Thermo Scientific; Cat.No: 78429). Lysates were homogenized, and protein concentrations were quantified using the enzymatic BCA Protein Assay (Thermo Scientific; Cat.No: 23227). SDS sample buffer (4× Laemmli Sample Buffer, BioRad, Feldkirchen, Germany, Cat.No: 1610747) containing TCEP Bond Breaker (Thermo Scientific, Cat.No: 77720) was added, but probes were not heat denatured. 20 µg of protein per lane were separated on 10% SDS-polyacrylamide gels (BioRad, Feldkirchen, Germany, Cat.No: 1610158) by electrophoresis.

Proteins were transferred to Immobilon-P PVDF membranes (Merck Millipore, Burlington, MA, USA) for immunoblotting. Membranes were blocked with 5% nonfat dry milk (BioRad, Feldkirchen, Germany, Cat.No: 1706404) in Tris-buffered saline (TBS: 140 mM NaCl, 10 mM Tris-HCl, pH 7.4) and incubated with a CXCR4-specific primary antibody (Abcam, Cambridge, UK, Cat.No: ab124824) and the loading control ß-actin (Santa Cruz Biotechnology, Dallas, USA, Cat.No: sc-47778-HRP) for 2 h at room temperature or overnight at 4 °C. After extensive washing with TBS, membranes were incubated with anti-rabbit IgG secondary antibody (Promega Corporation, Madison, WI, USA, Cat.No: W4011) for 1 h. Protein detection was performed using an enhanced chemiluminescence system (ECL Clarity Western ECL Substrate, BioRad, Feldkirchen, Germany, Cat.No 1705061), with molecular weight markers (Precision Plus Protein Standards; Cat.No 1610375) included. Band intensities were analyzed using GelAnalyzer 23.1.1 software^19^. CXCR4 signals were first normalized to ß-actin to control for loading differences, and subsequently normalized to the medium-treated control for each experiment to allow comparison between treatment conditions. Three independent biological replicates were performed, and the resulting normalized values were used for statistical analysis.

### CXCR4 receptor uptake

BON-1, QGP-1, and MS-18 cells were seeded in 24-well plates (Greiner Bio-One B.V., Kremsmünster, Austria) at a density of 2 × 10^5^ cells per well and incubated with the respective systemic therapeutics under the same conditions described above. Following defined incubation times, the supernatant was removed, and three wells per treatment group were incubated with 500 µl PBS containing approximately 50 kBq [^68^Ga]-Pentixafor for 1 h at 37 °C. After tracer incubation, the cells were washed once with ice-cold PBS and detached by trypsinization. The cell suspensions were transferred to Eppendorf tubes and washed twice more with ice-cold PBS. Radioactivity was measured using a Wizard^2^ 2480 gamma counter (Perkin Elmer, Waltham, MA, USA). Cell numbers were subsequently determined to standardize uptake values. Tracer uptake was expressed as the percentage of the initial administered radioactivity per 1 × 10^6^ cells.

### Statistics

Statistical analyses were performed using Microsoft Excel (Microsoft, Redmond, Washington, USA) and GraphPad Prism (version 10.4.2, GraphPad Software, Boston, Massachusetts, USA). Data normality was assessed using the Shapiro-Wilk test. For normally distributed datasets, comparisons between groups were assessed using the unpaired Student’s *t*-test. A *p*-value of < 0.05 was considered statistically significant. Data are presented as mean ± standard deviation (SD).

## Results

### Effects of the system therapeutics on CXCR4 expression

As shown in previous research, the basal CXCR4 expression was considerably higher in our MS-18 cell line compared to the BON-1 and QGP-1 cell lines^16^. qRT-PCR analysis revealed that etoposide treatment did not significantly alter CXCR4 mRNA expression in any of the cell lines. After 24 h of streptozotocin exposure, a significant decrease in CXCR4 mRNA was observed in BON-1, while QGP-1 and MS-18 showed no significant changes. Cisplatin treatment for 48 h led to a significant reduction of CXCR4 mRNA in BON-1 and QGP-1, with MS-18 remaining largely unaffected. Exposure to 5-FU significantly decreased CXCR4 mRNA levels in QGP-1 and MS-18, whereas BON-1 expression was unchanged. Everolimus treatment resulted in a significant decrease in CXCR4 mRNA in BON-1 and QGP-1, but not in MS-18. Finally, temozolomide significantly reduced CXCR4 mRNA expression in all three cell lines.

**Fig. 1:**
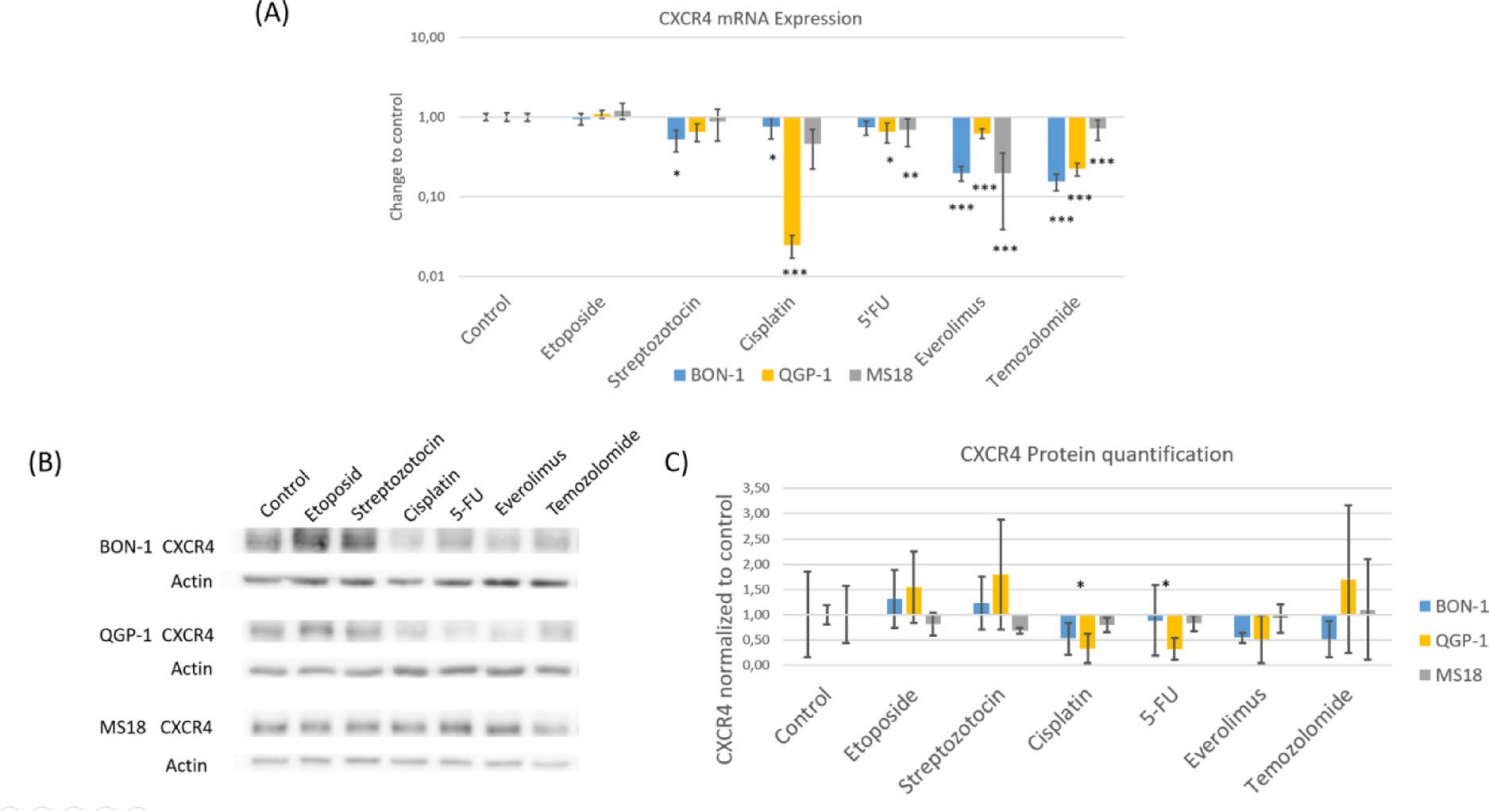
**(A)** The CXCR4 mRNA expression in the cell lines BON-1, QGP-1, and MS-18 after incubation with cisplatin, etoposide, streptozotocin, 5-fluorouracil, temozolomide, and everolimus in relation to control. The expression levels were normalized to housekeeping gene GAPDH (mean ± SD; *n* = 6 experimental repeats; * *p* < 0.05, ** *p* < 0.01, ***p < 0,001). **(B)** CXCR4 protein in Western Blot illustrated with ß-actin as loading control. **(C)** CXCR4 protein quantification of the western blot with medium control (*n* = 3, * *p* < 0.05).CXCR4 immunochemistry was performed on all cell lines after incubation with the study compounds (Fig. 2). As observed in the Western blot expression analysis, the MS-18 cells showed stronger CXCR4 staining compared to the BON-1 and QGP-1cell lines. In MS-18, a strong expression was detected both on the membrane and in the nucleus, corresponding to examination of the distribution of CXCR4 in the cell with a cell compartment kit (data not shown). The intensity of the staining was diminished following exposure to cisplatin, 5-FU, everolimus, and temozolomide, which corroborates the observations from qRT-PCR and Western Blot. In QGP-1 and BON-1 cells, no relevant changes in CXCR4 expression were observed, likely due to the low basal expression in these cell lines.

In Western blot quantification, only in BON-1 after incubation with cisplatin and 5-FU, a significant decrease in protein expression was detected. Western blot quantification revealed a significant decrease in CXCR4 protein only in BON-1 after incubation with cisplatin (*p* = 0,0299) and 5-FU (*p* = 0,0150). In the other treatments, including etoposide, streptozotocin, everolimus, and temozolomide, no statistically significant changes were observed, although a trend toward reduced protein expression was apparent in BON-1 and QGP-1 for cisplatin, 5-FU, everolimus, and temozolomide, reflecting the general decrease observed at the mRNA level (Fig. 1).

**Fig. 2:**
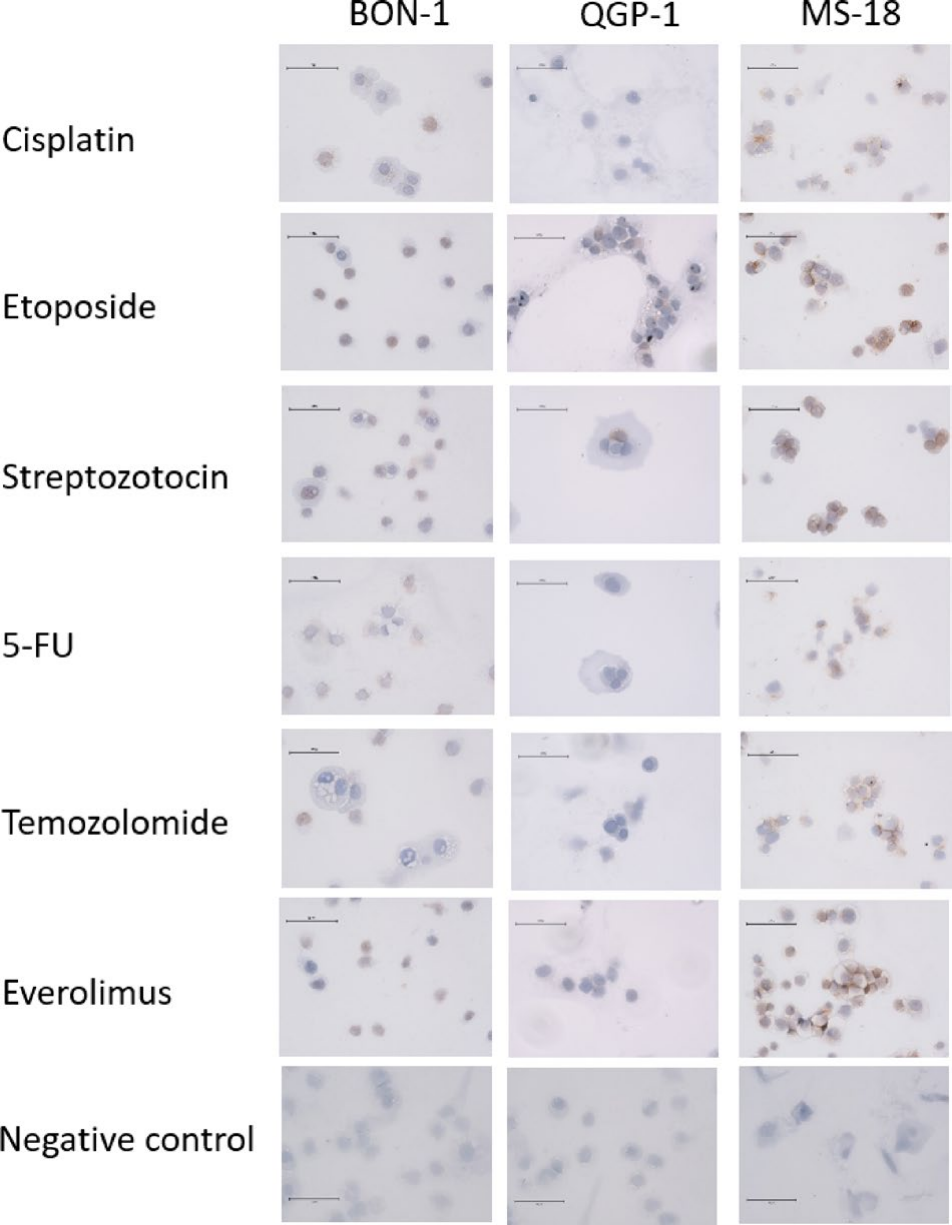
Immunohistochemistry staining of CXCR4 in BON-1, QGP-1, and MS-18 cells after incubation with the systemic therapeutics as well as medium control. The bar in the images represents 75 μm.

### Effects of systemic therapeutics on [^68^Ga]Pentixafor uptake

**Fig. 3:**
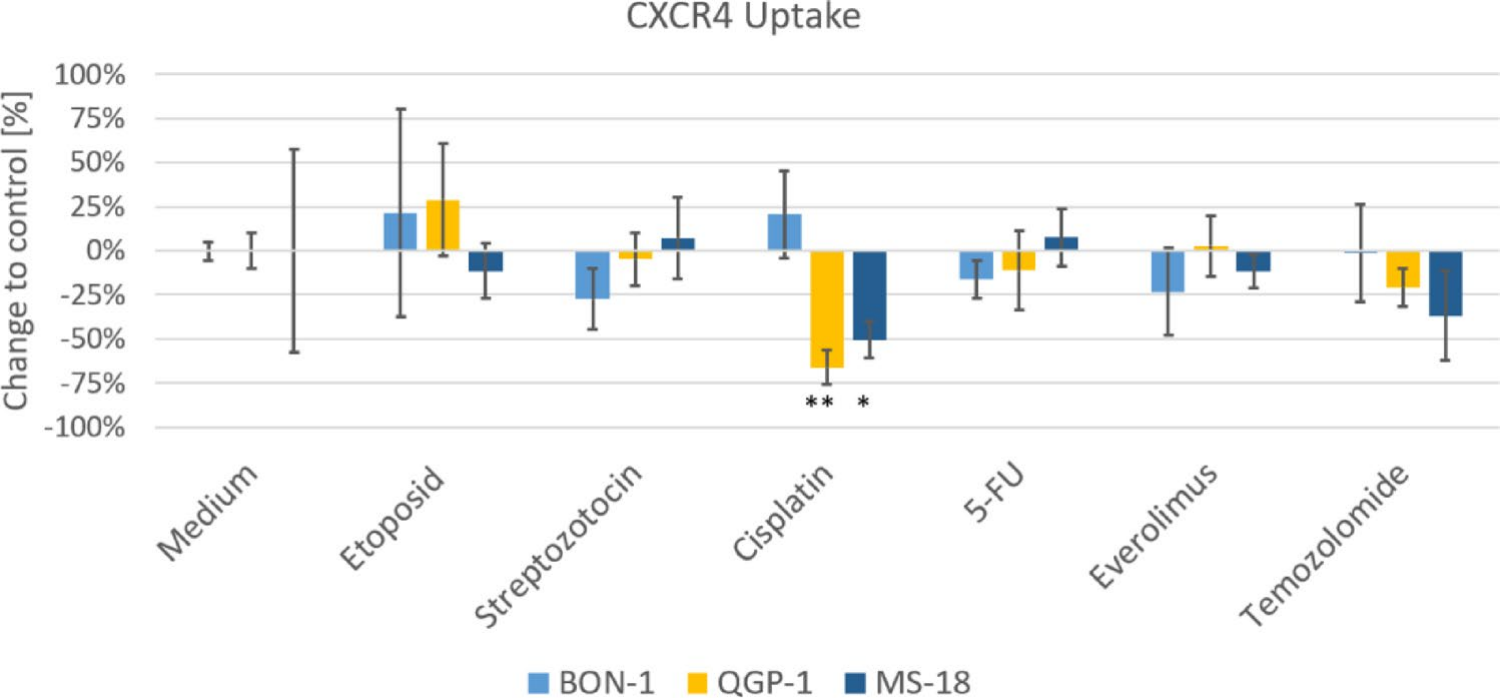
Specific uptake of radiolabeled [Ga68] Pentixafor in BON-1, QGP-1, and MS-18 cells after incubation with the systemic therapeutics. The data are presented as percentual change to untreated controls (mean ± SD; *n* = 3; * *p* < 0.05, ** *p* < 0.01).

The cells from the three used cell lines were incubated with the PET tracer [^68^Ga]Pentixafor, a CXCR4-prefering ligand, both with and without prior exposure to the systemic therapeutics. The effects of the different drugs on uptake are shown in Fig. 3. A significant effect on [^68^Ga]Pentixafor uptake was only observed after exposure to cisplatin, resulting in a decrease in both QGP-1 and MS-18. This result aligns with the decreased CXCR4 expression at the mRNA and protein levels in QGP-1. The decrease in CXCR4 expression at the mRNA and protein levels seen after 5-FU, temozolomide, and everolimus did not manifest in a significant effect on CXCR4 receptor uptake, although there was a trend towards decreased uptake. However, this trend was not statistically significant.

## Discussion

Treatment of patients with differentiated NET using somatostatin analogs or peptide receptor radionuclide therapy (PRRT) depends on the presence of SSTR2 expression across all tumor sites.

However, as tumors undergo dedifferentiation, such as in NEC, this tissue-specific receptor is frequently lost. These immunohistochemical findings correlate with a loss of tracer uptake in [^68^Ga]DOTATOC-PET/CT, highlighting the urgent need for alternative therapeutic targets in high-grade NENs lacking SSTR expression. The increased aggressiveness and metastatic potential seen in these dedifferentiated tumors may partly be attributed to elevated CXCR4 expression. This inverse correlation between SSTR2 and CXCR4 has been observed in NEN both histologically and through functional in vivo imaging^4,6^.

Overactivation of the CXCR4/CXCL12 axis is associated with larger tumor size, greater dedifferentiation, higher incidence rates, reduced response to cytostatic therapy, and decreased overall survival across various entities^10,20^. As mentioned above, ongoing trials are exploring the potential of CXCR4 in oncology through the use of inhibitors and CXCR4-targeted antibodies^10,11^.

In this context, CXCR4 has emerged as a promising target for both therapeutic and imaging applications in hematological and solid tumors^21^. Imaging of CXCR4 using [^68^Ga]Pentixafor PET/CT has demonstrated the ability to detect immunohistochemically positive lesions in dedifferentiated tumors. However, due to its lower sensitivity compared to [^18^F]FDG PET/CT, the latter remains the preferred imaging modality for dedifferentiated NEN at present^6,7,22^. Despite this, CXCR4-positive tumor volume assessed by imaging has been shown to predict survival outcomes in patients with high-grade NEN^23^. Furthermore, systemic therapies commonly administered to these patients may reduce CXCR4 expression, potentially leading to diminished diagnostic readouts, as CXCR4 expression appears to be treatment-sensitive. Therefore, a deeper understanding of how these treatments affect CXCR4 expression is essential for advancing this imaging approach.

In our study, we evaluated the impact of six systemic therapies commonly used in high-grade NENs. Following incubation with cisplatin, one cell line exhibited a significant reduction in CXCR4 expression, while the other two showed more moderate changes, likely reflecting the inherent heterogeneity of NENs. Additionally, decreased CXCR4 expression correlated with reduced [^68^Ga]Pentixafor uptake in two cell lines after cisplatin treatment. Etoposide and streptozotocin caused only minor alterations in CXCR4 expression levels, whereas 5-FU induced significant changes. Notably, temozolomide and everolimus led to a significant downregulation of CXCR4 at both mRNA and with complementing trends on protein level in most cell lines. The changes in western blot quantification did not reach significance due to relatively high standard deviations. This pattern was consistent with [^68^Ga]Pentixafor uptake following treatment with these agents.

Platinum-based chemotherapy combined with etoposide remains the standard first-line therapy for NECs. The reduction of CXCR4 expression following cisplatin exposure may represent one mechanism of action of platinum-based treatment, as decreased CXCR4 levels are associated with lower metastatic potential and reduced tumor aggressiveness. In our study, etoposide showed no impact on CXCR4 expression. While streptozotocin statistically affected CXCR4 expression only in BON-1, 5-FU altered the expression in both QGP and MS18 cells. However, given the limited effect size, further research is needed. Both drugs are primarily used in lower proliferative pancreatic NENs. MS-18 is a hybrid cell line exhibiting features of NEC and NET G3, whereas BON-1 and QGP-1, originally classified as differentiated NET, have acquired mutations over prolonged passage - such as loss-of-function p53 mutations - resulting in NEC-like characteristics^24^. Temozolomide, a systemic therapeutic for highly proliferative NENs, was shown to reduce CXCR4 expression to a greater extent than streptozotocin and 5-FU. The reduced CXCR4 after incubation with everolimus may complement a previously described mechanism. Specifically, in NENs, the PI3K/Akt/mTOR pathway is upregulated via enhanced CXCR4-CXCL12 signaling, while mTOR inhibitors block downstream CXCR4 signaling effectors^25^. This supports the therapeutic use of mTOR inhibitors in NENs^5^. Thus, the reduction in CXCR4 expression may contribute to the efficacy of everolimus, a phenomenon recently also reported in breast cancer^26^.

Currently, a variety of CXCR4-targeted therapeutic approaches are under development, including several small-molecule and peptide inhibitors, as well as the CXCR4-directed antibody ulocuplumab^10^. With a diagnostic and therapeutic radioligand tracer already approved for human use, the development of a theranostic approach seems promising. Encouraging results have already been reported in hematologic malignancies, such as diffuse large B cell lymphoma or multiple myeloma^21^. Since the NETTER-1 trial, SSTR2-directed endoradiotherapy has become an established treatment for NENs^27^. However, to date, for CXCR4-directed therapies in NEN, evidence is currently limited to preclinical animal studies^28^. Our data indicate that combining CXCR4-targeted therapy with any of the systemic agents tested in this study is unlikely to yield synergistic effects. As these drugs either reduced CXCR4 expression or had no measurable impact, an enhancement of CXCR4-targeted therapeutic efficacy cannot be expected. Moreover, the observed trend towards reduced [^68^Ga]Pentixafor uptake suggests that combining CXCR4-targeted endoradiotherapy with these compounds may not be beneficial. However, since a high CXCR4 expression correlates with a poorer prognosis and a more aggressive tumor phenotype, the CXCR4 downregulation by these systemic treatments may still be clinically advantageous in NENs. At the same time, if CXCR4-targeted diagnostics or therapies are planned, temporary discontinuation of such agents might be considered to preserve CXCR4 expression and maximize the effectiveness of the CXCR4-directed approach. The findings presented here may be relevant to guide the design and planning of clinical trials investigating CXCR4-directed approaches, which should ideally be investigated at stages where CXCR4 expression is highest.

Based on our findings, we also may hypothesize that the reduction of CXCR4 expression in NENs induced by systemic therapy may represent one mechanism underlying treatment efficacy. This hypothesis is supported by the effects of CXCR4 antagonists mitigating metastasis in an mouse model of NSCLC^10,29^. To validate this hypothesis, further preclinical in vivo data are needed. Variations in baseline CXCR4 expression in high-grade NENs could further serve as a prognostic marker, aiding in the prediction of remission onset and duration. While post-treatment tissue analysis does not seem feasible as sampling is usually only performed in case of disease progression, CXCR4 imaging with [^68^Ga]Pentixafor, both at baseline and after therapy induction, might be a useful tool for monitoring this endpoint. Thereby, our study results are not only relevant for the timing of an envisioned CXCR4-targeted therapy but also highlight potential advantages of certain agents that reduce CXCR4 expression for their antineoplastic effects.

One limitation of our study is the low number of available human NEN cell lines, a common challenge in this research field. Although BON-1 and QGP-1 are widely used models, they exhibit relatively low baseline CXCR4 expression^16,24^. To overcome this, we included the previously characterized hybrid NEN cell line MS-18, which combines features of both NET G3 and NEC and demonstrates higher CXCR4 expression^16^. While the use of MS-18 represents a strength, its detailed molecular characterization and mutational analysis have been reported elsewhere^16^. This inclusion improves the biological relevance and diversity of our in vitro model system. Another limitation is the lack of in vivo studies, which are necessary to confirm CXCR4 modulation by systemic therapies and evaluate clinical translation. Finally, while our study focused on clinically used pharmacologic treatments, we did not test CXCR4-targeted therapies directly, and the underlying genetic and epigenetic mechanisms driving differential responses across cell lines remain to be fully elucidated.

A major strength of our study is the use of a radionuclide-based internalization assay, which not only measures receptor expression but also reflects functional receptor activity and radiotracer uptake—providing a more comprehensive evaluation of CXCR4 dynamics.

To our knowledge, this is the first study to investigate therapy-induced alterations in CXCR4 expression and function in NEN cell lines, highlighting the novelty and relevance of our findings. Nonetheless, our research provides important preliminary data that contribute to understanding the potential of CXCR4 as a therapeutic target in NEN under systemic therapy.

## Supplementary Information

Below is the link to the electronic supplementary material.

Supplementary Material 1

## Data Availability

This article features the authors’ own research data which is available from the corresponding author upon request.
