# Supplementary material for: Impact of neuroendocrine neoplasm-specific systemic treatments on expression and function of CXCR4 in neuroendocrine tumor cells
Source: Sci Rep. 2026 Jan 31;16:4339. doi: 10.1038/s41598-026-37026-8 (PMC12864845; doi:10.1038/s41598-026-37026-8)

Supplementary information

Western Blots:

Cisplatin und Etoposide

QGP-1

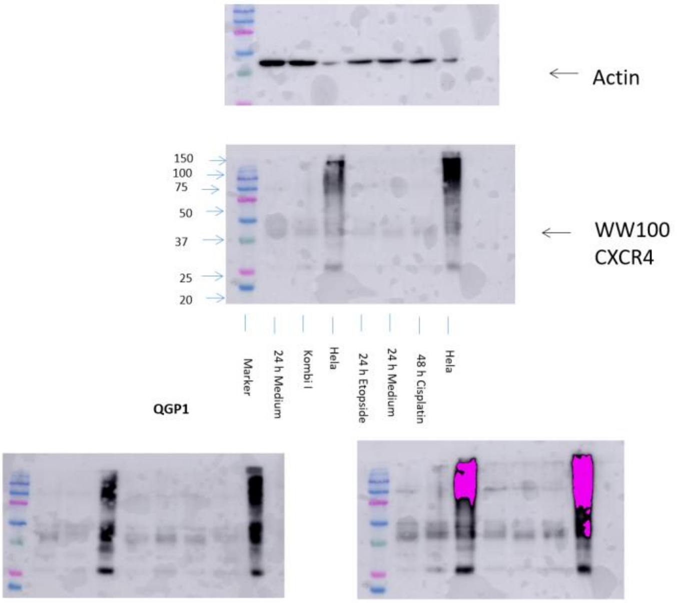

Cisplatin und Etoposide

QGP-1

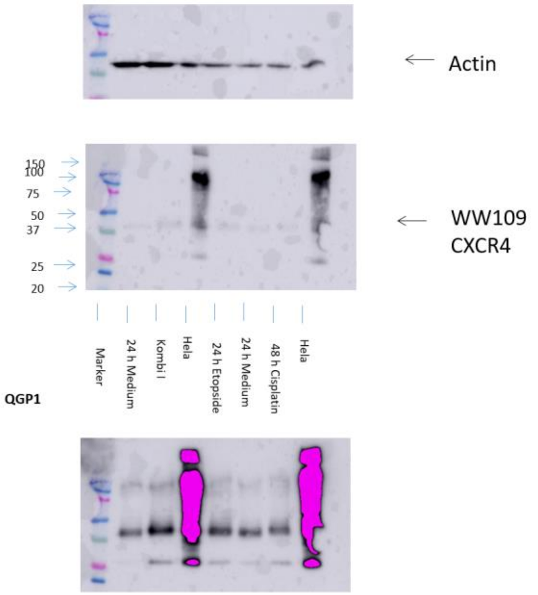

5-FU und Temozolomide QGP-1

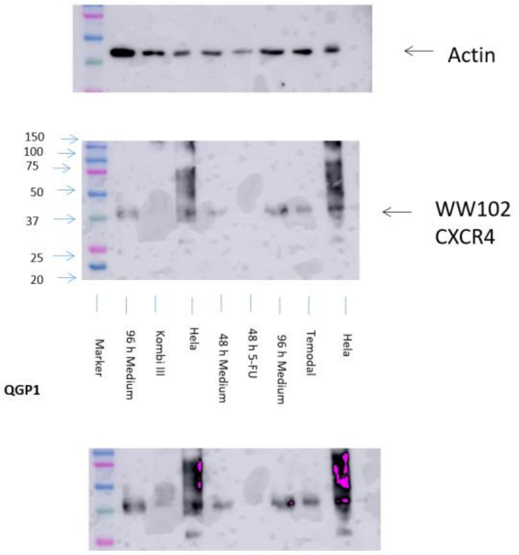

Everolimus QGP-1 und BON-1

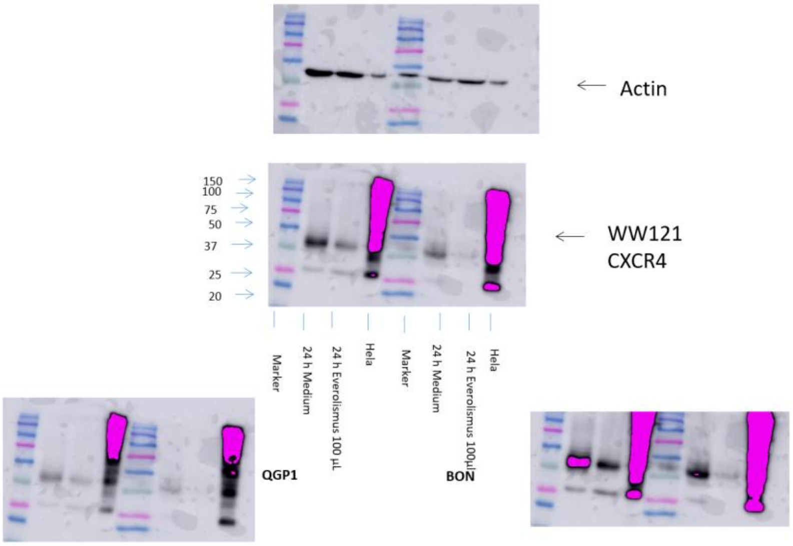

Cisplatin BON-1

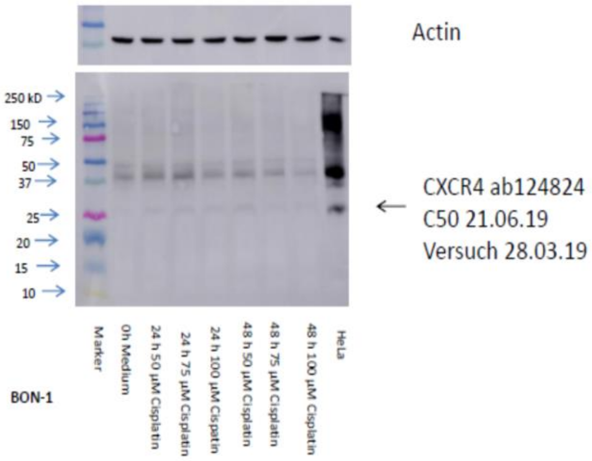

Cisplatin und Etoposide BON-1

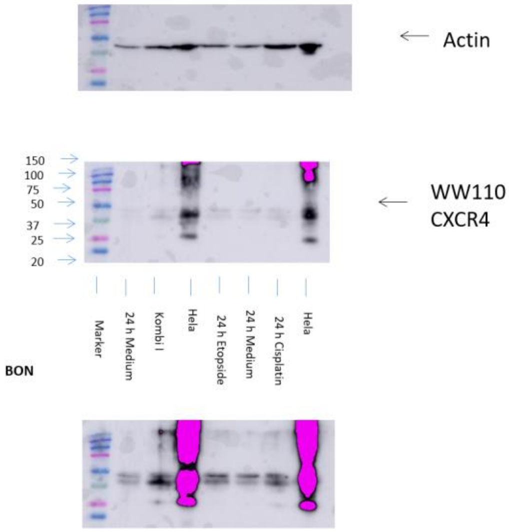

Streptozotocin und 5-FU

BON-1

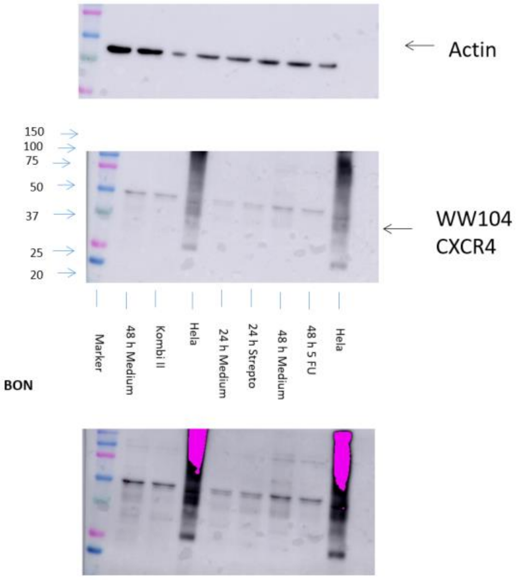

Everolimus

MS-18

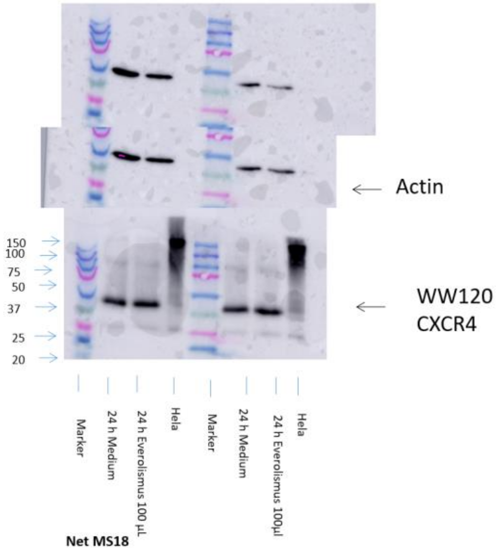

Cisplatin und Etoposide

MS-18

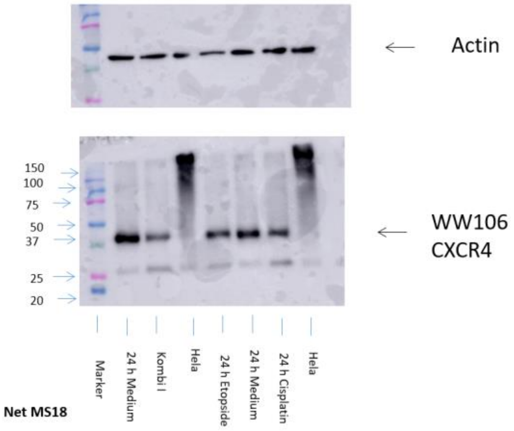

Streptozotocin und 5-FU

MS-18

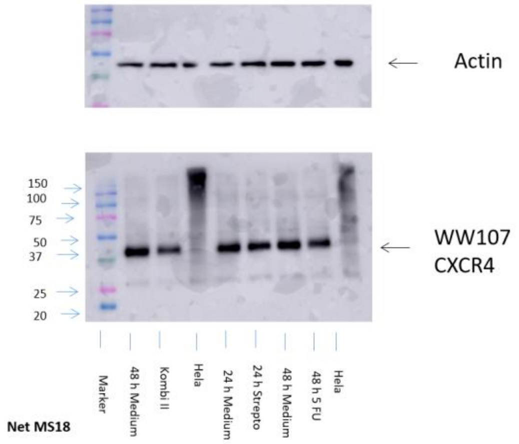

5-FU und Temozolomide

MS-18

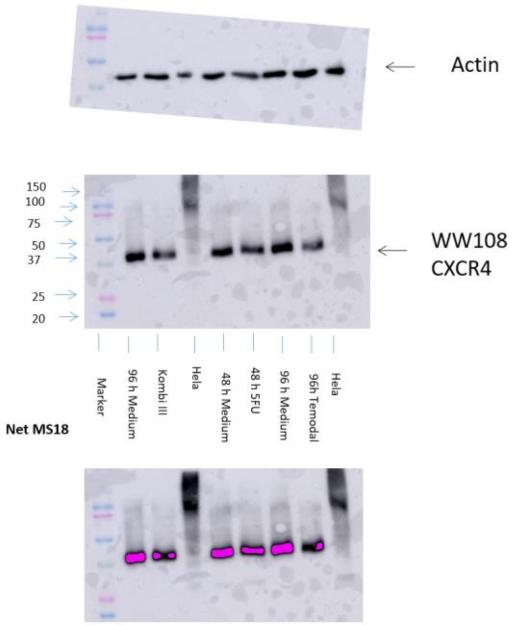

Supplement: Supplementary file 1 — Supplementary Material 1 [file 41598_2026_37026_MOESM1_ESM.pdf]
